# Tunable Natural and Magnetic Circularly Polarized Luminescence in the UVB Region from a Molecular Gd(III) Complex

**DOI:** 10.1021/jacsau.6c00463

**Published:** 2026-05-23

**Authors:** Luca Gherardi, Alessio Gabbani, Lorenzo Di Bari, Francesco Zinna

**Affiliations:** Dipartimento di Chimica e Chimica Industriale, University of Pisa, Via Giuseppe Moruzzi 13, Pisa 56124, Italy

**Keywords:** CPL, MCPL, lanthanides, supramolecular, high energy emission, chiral macrocycles

## Abstract

Chiro-optical materials operating in the ultraviolet-B (UVB) region have the potential to enable transformative advances in asymmetric photocatalysis and covert security features. Nevertheless, molecular emitters with strong UVB circularly polarized luminescence (CPL) remain virtually nonexistent. We report a chiral Gd­(III) complex that fills this gap with a tunable CPL at 312 nm. Molecular design takes advantage of a nonchromophoric chiral macrocyclic ligand promoting chiro-optical activity while avoiding competitive ligand absorption. The monomeric complex exhibits a sharp, high-quantum-yield emission (25%) with a CPL dissymmetry factor (*g*_lum_) up to 0.019. This CPL signal is dramatically enhanced and modified upon controlled dimerization, yielding a 10-fold increase in *g*_lum_. Furthermore, the system responds to magnetic fields, displaying unusually strong magnetic CPL (MCPL), which allows for the active modulation and reversal of the emergent polarization of the emission. The combination of high-energy emission, exceptional dissymmetry, and dual (chemical and magnetic) stimuli responsiveness positions these Gd­(III) complexes at the forefront of emerging UV chiro-optical technologies.

## Graphical abstract

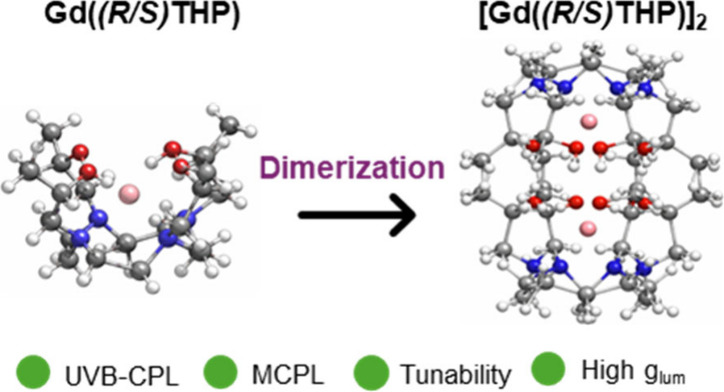

Circularly polarized luminescence (CPL) continues to evolve as a foundational spectroscopic tool, enabling breakthroughs in chiral electronics and photonics^1,2^ and bioimaging,^3–6^ as well as in the development of advanced security inks and labels.^7,8^ Current advancements in the field are driven by a dual focus: the design of compact, affordable instrumentation^8–16^ and the development of versatile CPL-active compounds.^17–22^ Particular interest lies in systems featuring stimuli-responsive CPL signals^23–30^ —modulated by molecular association or structural changes—as well as a modulation of the emergent CPL signal through magnetic fields.^31–37^ Moreover, these functional characteristics should be complemented by the design of new CPL-materials that span an increasingly broad range of the optical electromagnetic spectrum.^38^

In the last years, substantial efforts have been devoted to extending CPL emitters beyond the visible. A few systems were recently developed showing highly polarized emission in the NIR, based on chiral coordination compounds^39^ (mainly Cr­(III),^40–43^ Yb­(III),^44–52^ and Er­(III)^53–59^ ) and even purely organic materials.^60,61^ On the other hand, examples extending CPL emitters on the higher energy side of the electromagnetic spectrum, and especially into the UVB region (290–320 nm) are almost unexplored.^62^ Only very recently, Long et al.^63^ reported the first UVB CPL, observed from a Gd-based organic–inorganic hybrid metal halide, with a CPL dissymmetry factor (*g*_lum_) in the order of 10^–3^. Despite these recent efforts, chiral molecular emitters with CPL in the high energy region of the spectrum are still lacking. UVB CPL emitters may find applications in UV-CP-OLEDs, in asymmetric photocatalysis and invisible security inks or labels.^8,64^ In the specific case of Gd, UVB emission is used to detect Cherenkov radiation from cosmic muons,^65^ or, in principle, to quantify Gd species in plasma, after its use as an MRI contrast agent.^66^

The main emission band in Gd^3+^ is associated with the ^6^P_7/2_↔^8^S_7/2_ transition occurring at 311–312 nm (∼32 100 cm^–1^ or ∼3.98 eV).^67^ Similarly to other emitting lanthanides,^64,68–70^ if Gd ion is encapsulated in a proper chiral environment, significant CPL could be expected. Such transition entails Δ*S* = 1, Δ*L* = 1, Δ*J* = 0. Therefore, according to Richardson’s theory, it belongs to class II in a I–IV scale (with I > II > III > IV) for oscillator (E) and rotatory (R) strength and dissymmetry (D).^71^ Therefore, ^6^P_7/2_↔^8^S_7/2_ transition is expected to show moderately strong circular dichroism and CPL with potentially high absorption and emission dissymmetry factors. On the other hand, several common lanthanide ligands bear conjugated chromophores which strongly absorb in the UVB region, making unpractical or impossible to observe such transition.

Here, we report, for the first time, a strong CPL emission (Figure 1b) around 312 nm from a chiral molecular Gd compound. The CPL signal can be tuned upon dimerization of the complex (Figure 1c). Moreover, this compound displays an unusually strong magnetic CPL (MCPL) activity, which can be used to modify, and even reverse, the emergent emission polarization. To this end, we employed a chiral THP (1,1′,1″,1‴-(1,4,7,10-tetraazacyclododecane-1,4,7,10-tetrayl)­tetrakis­(propan-2-ol)) macrocyclic ligand (Figure 1a). THP is based on the cyclen scaffold functionalized with chiral arms bearing OH groups.^72^ The resulting Gd­(THP) complex is structurally reminiscent of Gd–DOTA and its derivatives,^73–78^ commonly used as paramagnetic relaxation agents in MRI.^79,80^

**Figure 1:**
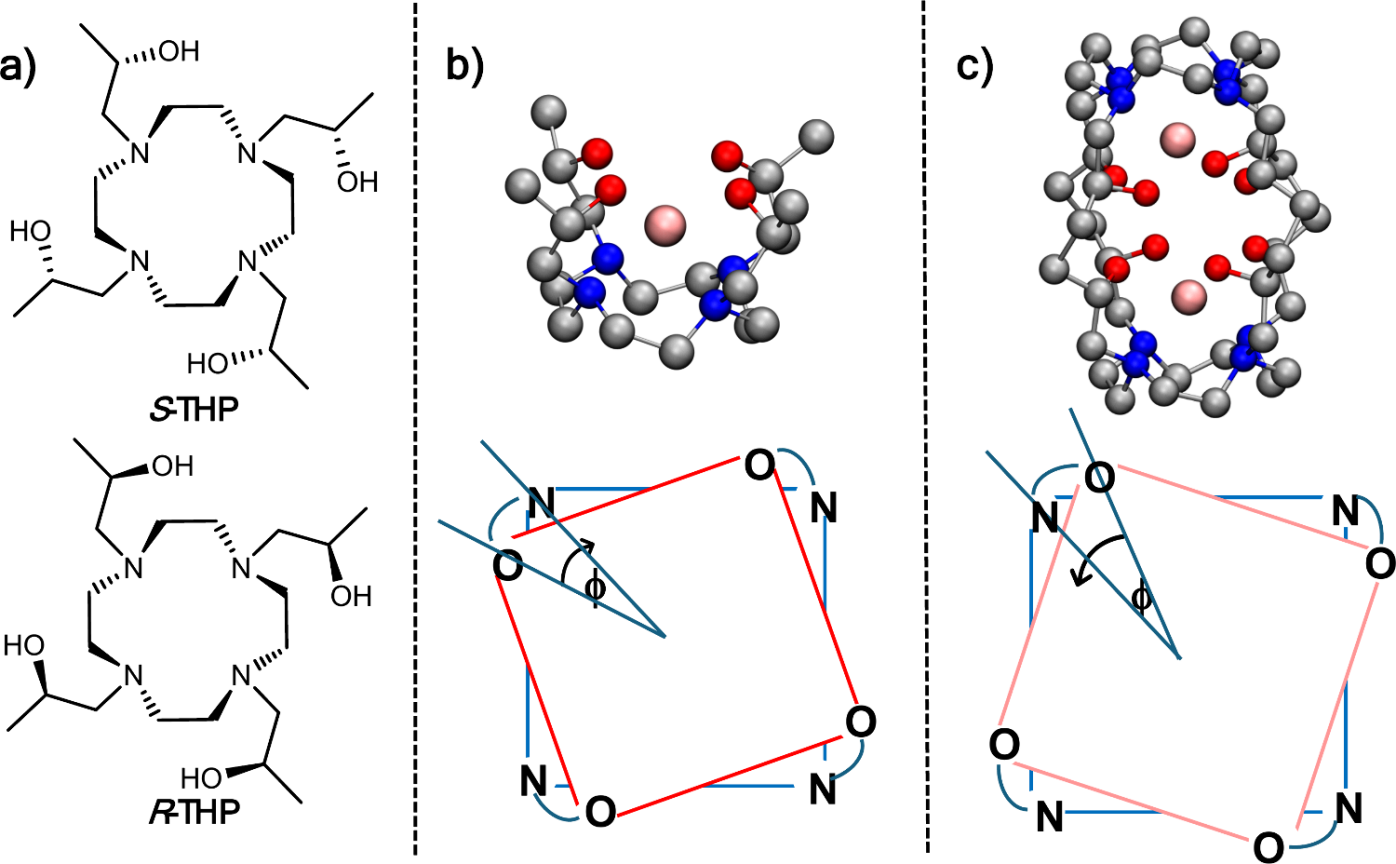
(a) Ligands employed in this study, (b) (top) side view and (bottom) schematic representation of the relative conformation of the ligand in the Gd­((*S*)­THP) monomeric species, (c) (top) side view and (bottom) schematic representation of the conformation of one monomeric unit within the dimeric structure [Gd­(*(S)*THP)]_2_. The structures are based on those reported for the isostructural Yb­(THP) complexes.^81^

Moreover, the corresponding Yb­(THP) analogues were extensively investigated from both spectroscopic and structural points of view in solution,^81^ even though single-crystal XRD analysis of enantiopure compounds was unfeasible. This ligand brings about two main advantages: (i) the absence of chromophore units that could bring significant absorption above 200 nm and (ii) the stereo-ordered arrangement of the arms around the metal center creating a twisted coordination polyhedron (Figure 1b). These features make this complex suitable for the observation of strong absorption and emission chiro-optical activity of Gd.

Both the enantiomers of Gd­(THP) complexes were prepared following a literature procedure, by reacting THP ligand with an equimolar amount of Gd­(OTf)_3_.^72^ The complexes were studied in acid aqueous solutions (see the Supporting Information for more details regarding the preparation and the analysis conditions, as well as an analysis of the spectral features as a function of pH). Under direct excitation of the ^6^I_7/2_→^8^S_7/2_ transition at 274 nm, a strong emission associated with the ^6^P_7/2_→^8^S_7/2_ manifold (311.7 nm), accompanied by a much weaker band associated with the ^6^P_5/2_→^8^S_7/2_ (306.1 nm) was observed (Figure 2c). The main band is remarkably sharp, featuring a full width at half-maximum of 0.6 nm (65 cm^–1^), with an emission quantum yield (QY) of approximately 25% with respect to naphthalene (QY = 23% in hexane). Interestingly, the ^6^P_7/2_→^8^S_7/2_ transition was associated with a bisignate CPL activity (Figure 2c) with the two peaks at 312.1 and 311.55 nm. Given the presence of these two opposite-signed bands, it is important to carry out the measurement with narrow emission slit widths (0.05 nm in our case), in order to avoid partial signal cancellation.^82^ The calculated luminescence dissymmetry factors at 312 nm (*g*_lum_) were 0.019 and 0.018 for the *S* and *R* enantiomer, respectively. Such values allowed us to calculate the so-called “CPL figure of merit” (FoM = *g*_lum_ × QY) on the order of 10^–3^, which is an order of magnitude higher than the value recently reported for the first UVB CPL active compound. Similarly, a CPL brightness^83^ (*B*_CPL_), calculated according to ref 39 of 1.3 × 10^–3^ (in the 310.0–311.8 nm range) and 2.8 × 10^–3^ (311.8–315.0 nm) cm^–1^ M^–1^ was estimated.

**Figure 2:**
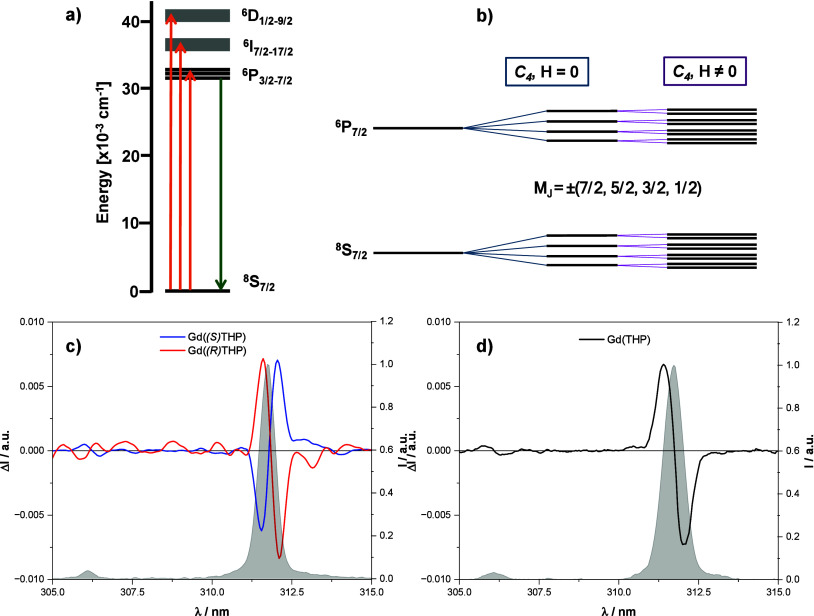
(a) Term-to-term transitions of Gd­(III); (b) splitting of the ground and emitting state in a C_4_-symmetry environment in absence and presence of a magnetic field; (c) CPL and (d) MCPL of Gd­((*R*)­THP) in aqueous solution (pH <1, *C* ≈ 9 mM). Total emission spectrum is traced in the background.

The CPL spectral shape was almost unaffected by changing the conditions, namely, increasing the pH of the aqueous solution (up to 10.6) or changing the solvent to acetonitrile (Figures S17 and S20).

It is important to note that the relatively simple CPL signature observed for this complex is the convolution of several transitions forming the manifold. In a C_4_ environment, both the ^6^P_7/2_ excited state and ^8^S_7/2_ ground state are split into 4 M_J_ nondegenerate Kramers doublets each (Figure 2b). Given that the whole manifold encompasses only 4 nm (∼400 cm^–1^), all the 4 doublets of the ^6^P_7/2_ term are significantly populated at room temperature (*k*_B_ *T* = 200 cm^–1^). This would yield up to 4 × 4 = 16 transitions. Upon applying a +1.6 T magnetic field (H), the emergent emission circular polarization of the bands is reversed for Gd­((*S*)­THP), and similarly a −1.6 T field reverses the polarization for Gd­((*R*)­THP) (Figure S1). The magnetic field induced CPL (MCPL) and the natural CPL can be disentangled by recording two CPL spectra under positive (CPL­[+]) and negative (CPL[−]) magnetic field (see Figure S3 in the Supporting Information).^84,85^ In this way,

1 $$\mathrm{MCPL}=\frac{1}{2}\left(\mathrm{CPL}\left[+\right]-\mathrm{CPL}\left[-\right]\right)$$

The MCPL spectrum (Figure 2b) shows again a bisignate band. It is possible to define a magnetic field-normalized MCPL dissymmetry factor as:

2 $$g_{\mathrm{MCPL}}=2\frac{1}{H}\frac{\left(I_{L}-I_{R}\right)}{\left(I_{L}+I_{R}\right)}$$

*g*_MCPL_ was calculated to be −0.032 T^–1^ at 312.0 nm and 0.038 T^–1^ at 311.4 nm. These values are on the higher side, compared to those typically reported for lanthanide MCPL.^86–93^ Interestingly, unlike what is commonly observed for chiral inorganic complexes, Gd­(THP) shows a MCPL signal that is significantly stronger than natural CPL, with a MCPL/CPL ratio of 1.21 for the 312 nm band and 1.9 for the 311 nm band. Again, this is the result of a convolution of several transitions. In a magnetic field, each Kramers doublet splits into a higher and lower energy component, separated by an energy difference on the order of 1 cm^–1^ T^–1^, giving up to 8 nondegenerate states for both the ground and excited states (16 in total, Figure 2b). Assuming that only transitions with Δ*M*_J_ = ±1 are allowed, up to 14 distinct transitions may take place.^94,95^ Expectedly,^96,97^ MCPL of Gd­((*S*)­THP) and Gd­((*R*)­THP) are exactly the same (see Figure S2).

Upon the addition of a base (Et_3_N) to a dry CH_3_CN solution of Gd­(THP), the dimeric [Gd­(THP)]_2_ is obtained, consistently with literature data and confirmed by high-resolution mass spectrometry (HRMS), which displays an isotopic pattern consistent with the presence of two Gd centers (Figure S3) rather than a single one as in the monomeric species (Figure S4). The formation of the dimeric structure was further investigated by monitoring the CPL signal upon addition of subsequent equivalents of triethylamine (Figure S18) in acetonitrile. In the absence of a base, only the CPL signal of the monomer is present (∼312 nm). Upon addition of Et_3_N a decrease in intensity of the peak at 312 nm is observed, accompanied by the emergence of a new peak at ∼313 nm, characteristic of [Gd­(THP)]_2_. The spectral shape reaches a plateau consistent with the presence of the dimeric species after 2 equiv of triethylamine (Figure S19).

[Gd­(THP)]_2_ shows a weaker but significant emission around 312 nm, associated again with ^6^P_7/2_→^8^S_7/2_. Its CPL shows a strongly asymmetric bisignate feature (Figure 3a), with a *g*_lum_ of 0.22 and 0.16 for [Gd­((*S*)­THP)]_2_ and [Gd­((*R*)­THP)]_2_, respectively, at 313.8 and 313.9 nm, where the more prominent contribution is observed. Such values are approximately an order of magnitude higher than those recorded for the monomeric complexes. Interestingly, the main bands of the dimer are opposite in sign, with respect to the monomeric Gd­(THP) (Figure 1). This is consistent with the ring inversion of the side arm conformation upon dimerization, as previously observed for the analogous Yb­(THP) complex.^75,77,78,98^ This rearrangement effectively transforms the Δ conformation of the coordination polyhedron in Yb­((*S*)­THP) into Λ in [Yb­((*S*)­THP)]_2_ (with the φ angle going from 15.1° to −16.6°, respectively; see Figure 1c).^81^ In contrast, MCPL showed a spectral shape similar to that of the monomeric Gd­(THP), with *g*_MCPL_ values in the same order of magnitude (0.018 T^–1^ at 311 nm) as the monomer. Interestingly, the dimerization and the resulting twist of the coordination polyhedron strongly affects CPL both in sign and intensity, but it has only minor effects on the MCPL response. This observation confirms that, although both techniques can be sensitive to structural rearrangements, magneto-optical techniques are less sensitive than chiro-optical ones to geometry variations and longer-range interactions.^99,100^ We recall, anyway, that magneto-optical techniques are not sensitive to absolute handedness.^96^

**Figure 3:**
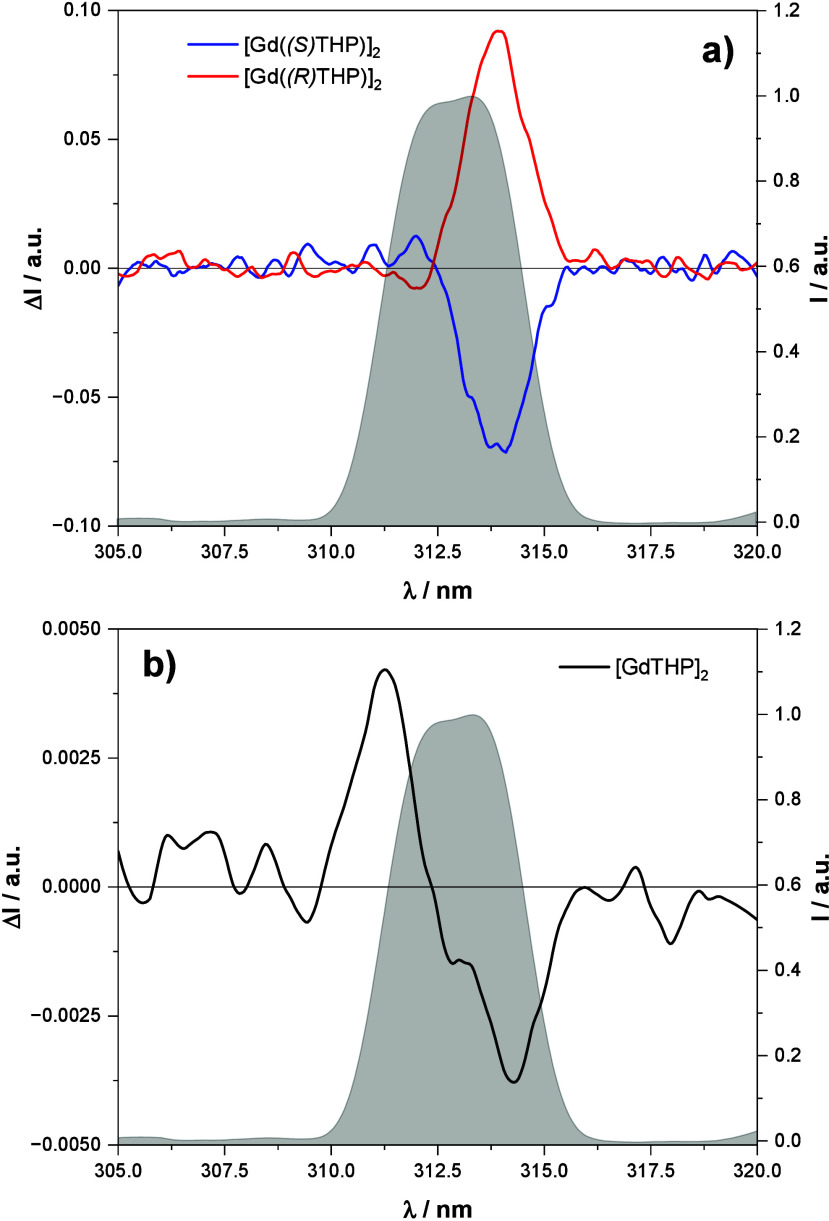
(a) CPL of [Gd­(THP)]_2_ and (b) MCPL of [Gd­((*S*)­THP)]_2_ recorded in acetonitrile (*C* ≈ 11 mM). Total emission spectrum is traced in the background.

Finally, thanks to the absence of chromophore groups on the ligand, electronic circular dichroism (ECD) and magnetic circular dichroism (MCD) of monomer and dimer Gd complexes were recorded (Figure 4). In the case of Gd­(THP), ECD spectra showed a bisignate ^8^S_7/2_→^6^P_7/2_ band (Figure 4a), whose shape and magnitude (*g*_abs_ = 0.025 at 311.5 nm and 0.041 at 306.3 nm) are in agreement with the CPL results. In fact, given the small spacing between M_J_ levels, their Boltzmann populations at room temperature allow for the observation of all the transitions among all M_J_ levels both in absorption (hot bands) and in emission.^39^ ECD signals of similar magnitudes were also observed for ^8^S_7/2_→^6^P_5/2_ transition (306–312 nm) and for the multiplets associated with ^8^S_7/2_→^6^I_J_ (265–280 nm) and ^8^S_7/2_→^6^D_J_ (255–240 nm, Figure 4b). For [Gd­(THP)]_2_, the sign of the main ^8^S_7/2_→^6^P_5/2_ multiplet is inverted for the same enantiomer with respect to the monomer, confirming again the effect of the inversion of the coordination polyhedron twist upon dimerization.

**Figure 4:**
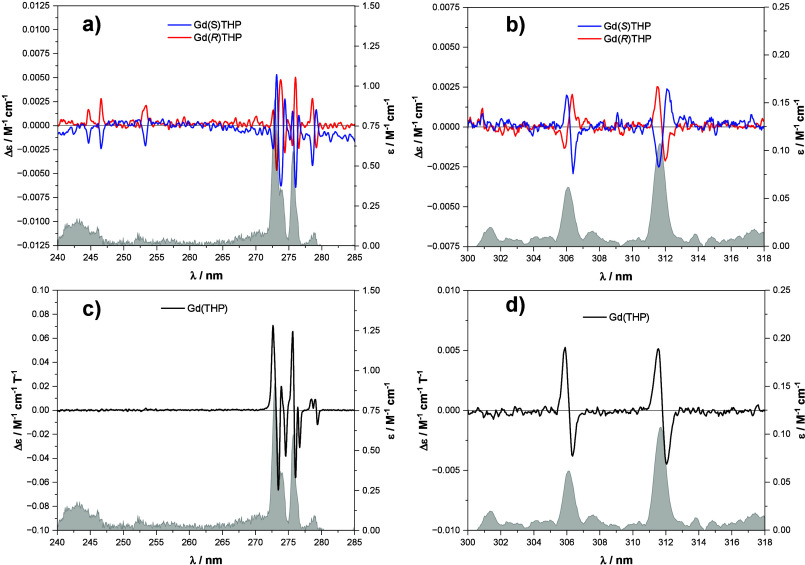
(a,b) ECD of Gd­(THP) and (c,d) MCD of Gd­((*R*)­THP) spectra in water (pH <1, *C* ≈ 27 mM). The corresponding absorption spectra are traced in the background.

Moreover, consistently with CPL results, [Gd­(THP)]_2_ shows ECD signal (Figures S7–S11), which are approximately an order of magnitude higher than those of the monomer (*g*_abs_ = 0.06 at 308 nm). MCD spectra of Gd­(THP) recorded for the ^8^S_7/2_→^6^P_5/2_ manifold is comparable in magnitude and shape to its respective MCPL bands (Figure 4c,d), and therefore the emergent circular dichroism spectrum is inverted upon application of +1.6 T magnetic field to Gd­((*S*)­THP) (see Figures S12–S14 in the Supporting Information).

In conclusion, we show highly circularly polarized luminescence in the UVB region associated with a narrowband emission of a chiral molecular complex. The same complex shows a remarkably strong MCPL, which can modify the sign of the emergent CPL response under a 1.6 T magnetic field. Finally, the CPL response is completely modified upon dimerization of the complex under basic conditions. These findings establish chiral Gd­(III) complexes as versatile platforms for UVB chiro-optical applications, addressing a nearly unexplored region of the electromagnetic spectrum. More broadly, this work demonstrates that lanthanide complexes are suitable as chiral photonic materials operating across the entire ultraviolet-to-infrared window.

## Supplementary Material



## Notes

Experimental details and methods: compound preparation and characterization, HRMS, additional spectra (PDF)
