# Supplementary material for: Tunable Natural and Magnetic Circularly Polarized Luminescence in the UVB Region from a Molecular Gd(III) Complex
Source: JACS Au. 2026 May 23;6(6):3114–21. doi: 10.1021/jacsau.6c00463 (PMC13291879; doi:10.1021/jacsau.6c00463)
Supplement: Supplementary file 1 [file au6c00463_si_001.pdf]

Tuneable natural and magnetic circularly polarized luminescence in the UVB region from a molecular Gd(III) complex

Luca Gherardi, Alessio Gabbani, Lorenzo Di Bari, Francesco Zinna*

Dipartimento di Chimica e Chimica Industriale, University of Pisa, Via Giuseppe Moruzzi 13, Pisa, Italy

francesco.zinna@unipi.it

Contents

1 General Experimental 3

1.1 Spectroscopy 3

2 Ligand and Complex General Procedures 5

2.1 Preparation of (R/S)-THP Ligand 5

2.2 Synthesis of Gd(R/S)THP monomeric species 6

2.3 Synthesis of Gd(R/S)THP dimeric species 7

3 Additional Figures 8

4 NMR Spectra 19

5 References 20

1 General Experimental

All reactions involving air sensitive compounds were carried out under N_2 via an inert gas/vacuum double manifold line and standard Schlenk techniques using dry solvents. All reagents were purchased at the highest commercial quality and used without further purification unless otherwise stated. NMR spectra were recorded on JEOL -500 ECZ and JEOL-400 ECZ, calibrated using residual undeuterated solvent ($CHCl_3$, MeOH, DMSO, MeCN, HDO at 7.26, 3.31, 2.50, 1.94, 4.79 ppm for ^{1}H NMR, respectively, and 77.16, 49.00, 39.52, 1.32 ppm for ^{13}C NMR, respectively).

1.1 Spectroscopy

ECD spectra were measured using a Jasco J–1500 spectropolarimeter in the range of 240-320 nm in H_2O (pH <1) in 1 cm quartz cells for the monomeric species of Gd(*S/R)*THP, on the other hand for the dimeric species [Gd(*S/R)*THP]$_2$ the solid was dissolved in acetonitrile (ACN) and recorded in the same spectral region as for the monomer. The spectra were background–corrected using solvent spectra recorded under the same conditions.

The acquisition parameters are for both the dimeric and monomeric species:

- Scan speed = 10 nm/min
- Data pitch = 0.05 nm
- Bandwidth = 0.5 nm
- Integration Time = 1sec

All MCD spectra were recorded with the same parameter as the ECD spectra using a 1.6 T Jasco Magnet.

CPL Spectra were measured using a Jasco- CPL300 in the range of 300-320 nm in H_2O (pH < 1) in 1 cm quartz cells for the monomeric species of Gd(*(S/R)*THP), on the other hand for the dimeric species [Gd(*(S/R)*THP)]$_2$ the solid was dissolved in acetonitrile (ACN) and recorded in the same spectral region as for the monomer.

The acquisition parameters are for both the dimeric and monomeric species:

- Scan speed = 10 nm/min
- Excitation Wavelength: 275 nm
- Data pitch = 0.05 nm
- Emission Bandwidth = 0.5 nm for Gd(THP) and 2 nm for [Gd(THP)]$_2$
- Excitation Bandwidth = 30.00 nm
- Integration Time = 1sec

All MCPL spectra were recorded with the same parameter as the ECD spectra using a 1.6 T Jasco Magnet.

Quantum yield

The fluorescence quantum yield (Φ_x) of the compounds was determined by the comparative method, using napthalene in hexane as the reference standard (Φ_{std} = 0.23). Emission spectra of both the sample and the standard were recorded under identical experimental conditions, using the same excitation wavelength (275 nm) and slit widths.

The quantum yield of the sample was calculated according to the following equation:

$$\Phi_x = \Phi_{std} \times \left(\frac{m_x}{m_{std}}\right) \times \left(\frac{n_x^2}{n_{std}^2}\right)$$

where:

- Φ_x and Φ_{std} are the quantum yields of the sample and the standard, respectively,
- m_x and m_{std} refer to the slopes of the linear fits obtained by plotting the integrated fluorescence intensities vs absorbance at the excitation wavelenght for the sample x and the standard respectively

$$m = \frac{Integrated\ Fluorescence\ Intensity}{Absorbance\ at\ excitation}$$

- n_x and n_{std} are the refractive indices of the solvents used for the sample and the standard.

HRMS Analysis

ESI-Q/ToF flow injection analyses (FIA) were carried out using a 1200 Infinity HPLC (Agilent Technologies, USA), coupled to a Jet Stream ESI interface (Agilent) with a Quadrupole-Time of Flight tandem mass spectrometer 6530 Infinity Q-TOF (Agilent Technologies). HPLC-MS grade acetonitrile was used as mobile phase (Carlo Erba, Italy). The flow rate was 0.2 mL/min (total run time 3 min). Injection volume: 4 µL.

The ESI operating conditions were: *drying gas* (N_2, purity >98%): 350 °C and 10 L/min; *capillary voltage* 4.5 KV; nozzle voltage: 1 KV; *nebuliser gas* 35 psig; *sheath gas* (N_2, purity >98%): 375 °C and 11 L/min. The fragmentor was kept at 50 V, the skimmer at 65 V and the OCT 1 RF at 750 V. High resolution MS and MS/MS spectra were achieved in positive mode in the range 100-1700 m/z; the mass axis was calibrated daily using the Agilent tuning mix HP0321 (Agilent Technologies) prepared in acetonitrile and water.

Sample preparation: 0.5 mg of each sample was weighted and diluted with 1 mL acetonitrile, filtered through PTFE filters (0.45 µm pore size) and diluted 500 times in acetonitrile.

PXRD

Powder x-ray diffraction was performed using a Anton Paar XRDynamic 500 diffractometer, equipped with Cu K-alpha radiation and working in Bragg Brentano geometry.

2 Ligand and Complex General Procedures

2.1 Preparation of (R/S)-THP Ligand

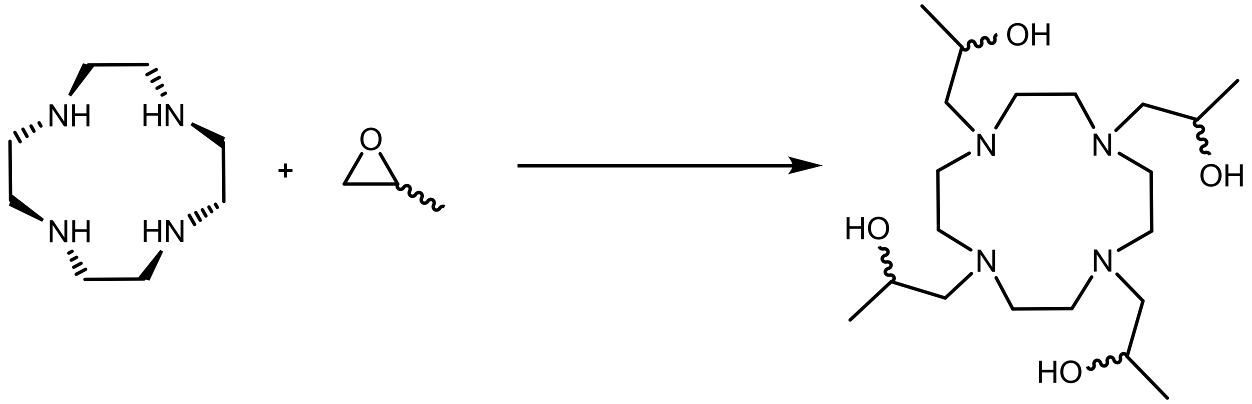

Following a literature procedure,[1] A 100 mL reactor with a magnetic stirrer bar and a nitrogen inlet, was charged with a mixture of Cyclen (2.46 g, 0.0143 mol, 1 equiv.) in ethanol (50 mL) and (R/S)-propyleneoxide (5 g, 0.086 mol, 6 equiv.) and it was left stirring for two days. Then upon evaporation a white solid was obtained. This solid was boiled in hexane, which was decanted from a sticky solid residue. White crystals were obtained from the cooled hexane solution by filtration.

Physical State: White Crystalline Solid

Isolated Mass for *R*-THP = 3.2 g, **Yield** = 55%

Isolated Mass for *S*-THP = 2.7 g, **Yield** = 48%

The NMR and characterization data for **(R/S)-THP** were consistent with previously reported NMR data.

^{1}H NMR (400 MHz, CD_3CN) δ 4.88 (s, 1H), 3.82 (dqd, *J* = 12.5, 6.2, 2.0 Hz, 1H), 2.99 – 2.85 (m, 2H), 2.32 (dd, *J* = 12.8, 10.2 Hz, 1H), 1.98 (dd, *J* = 12.8, 2.1 Hz, 1H), 1.95 – 1.84 (m, 2H), 0.97 (d, *J* = 6.3 Hz, 3H).

^{13}C NMR (126 MHz, CD_3CN) δ 63.4, 63.1, 62.5, 51.0, 47.9, 19.7

2.2 Synthesis of Gd(*(R/S)*THP) monomeric species

The lanthanide complexes were prepared, following the literature procedure reported for the complex of La, Eu, Yb and Lu. [1, 2]

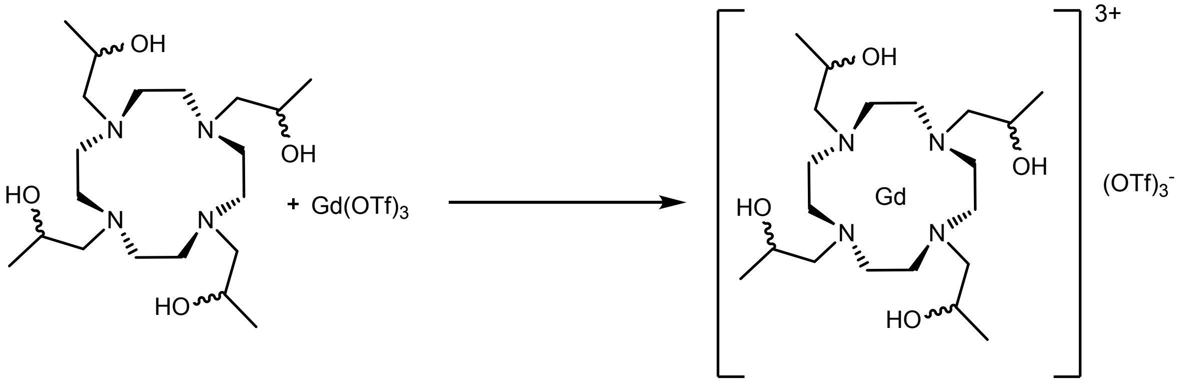

A 50 mL reactor with a magnetic stirrer bar, nitrogen inlet and a condenser was charged with a $Gd(OTf)_3$ (100 mg, 0.165 mmol, 1 equiv.) in dry ACN (7 mL), then 2/3 mL of triethyl orthoformate were added and the mixture was refluxed for 1 h. Then (R/S)-THP (66.7 mg, 0.161 mmol, 1 equiv.) was added to the flask and the mixture was refluxed for 1h. After 2h the mixture was evaporated, the crude product was crystallized from DCM/MeOH/Hexanes to obtain the title compound.

Physical State: White Crystalline Solid

Isolated Mass for Gd(*(R)*THP) = 88 mg, **Yield** = 53%

Isolated Mass for Gd(*(S)*THP) = 74 mg, **Yield** = 47%

Elemental Analysis: $C_{23}H_{44}F_9N_4O_{13}GdS_3 \cdot 1/3\ MeOH + 1/2\ H_2O$ Calcd.: C 27.24, H 4.54, N 5.45; Exp. C 27.23, H 4.51, N 5.43.

HRMS(ESI-qTOF, m/z) : calcd. For $[C_{20}H_{43}GdN_4O_4]^+$: 710.2046; Found: 710.2062

2.3 Synthesis of [Gd(*(R/S)*THP)]$_2$ species

The lanthanide complexes were prepared, following the literature procedure reported for the Yb complex.[2]

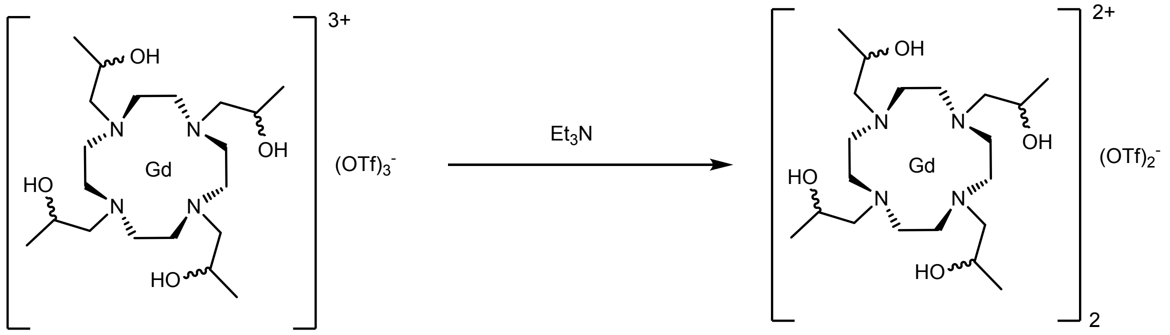

A 10 mL reactor with a magnetic stirrer bar, nitrogen inlet and a condenser was charged with either enantiomer of Gd(*(S/R)*THP) complex (50 mg, 0.05 mmol, 1 equiv.) in dry ACN (3 mL), then 4 equivalents of dry Et_3N were added and the mixture was stirred overnight. After 24 h the reaction mixture is evaporated, dissolved in DCM and precipitated with Hexane/Diethyl Ether to obtain the title compound.

Physical State: White Crystalline Solid

Isolated Mass for [Gd(*(R)*THP)]$_2$ = 40 mg, **Yield** = 57%

Isolated Mass for [Gd(*(S)*THP)]$_2$ = 43 mg, **Yield** = 61%

Elemental Analysis: $C_{42}H_{86}F_6N_8O_{14}Gd_2S_2 \cdot 1\ H_2O + 7/2\ DCM + 1/2$ Hexane. Calcd.: C 32.73, H 5.78, N 6.30; Exp. C 32.53, H 5.78, N 6.34.

HRMS(ESI-qTOF, m/z) : calcd. For $[C_{41}H_{85}Gd_2N_8O_{11}F_3S]^+$: 1269.4491; Found: 1269.4462

3 Additional Figures

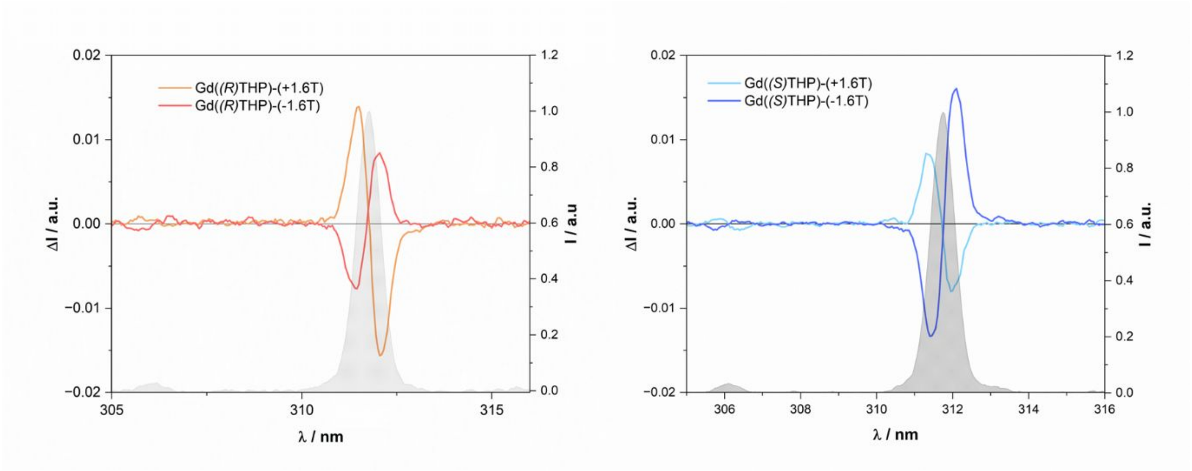

Figure S1. Superposition of the CPL and Emission spectra recorded in water (pH<1, C ≈ 9 mM) with both orientations of the magnet for Gd(*(S)*THP) and Gd(*(R)*THP) monomers.

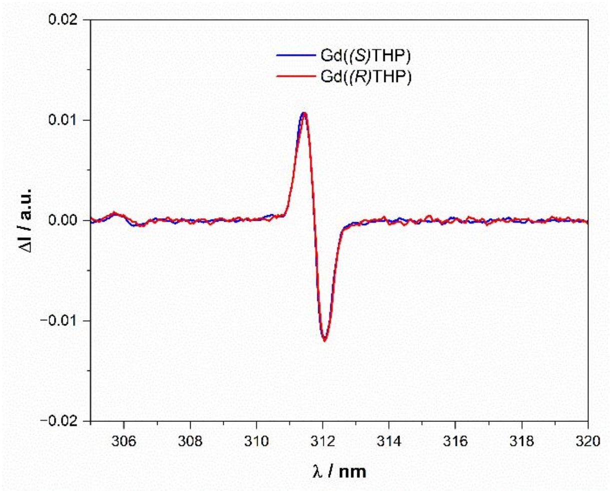

Figure S2. Superposition of the MCPL components for both enantiomers of Gd(THP) complexes recorded in water (pH<1, C ≈ 9 mM).

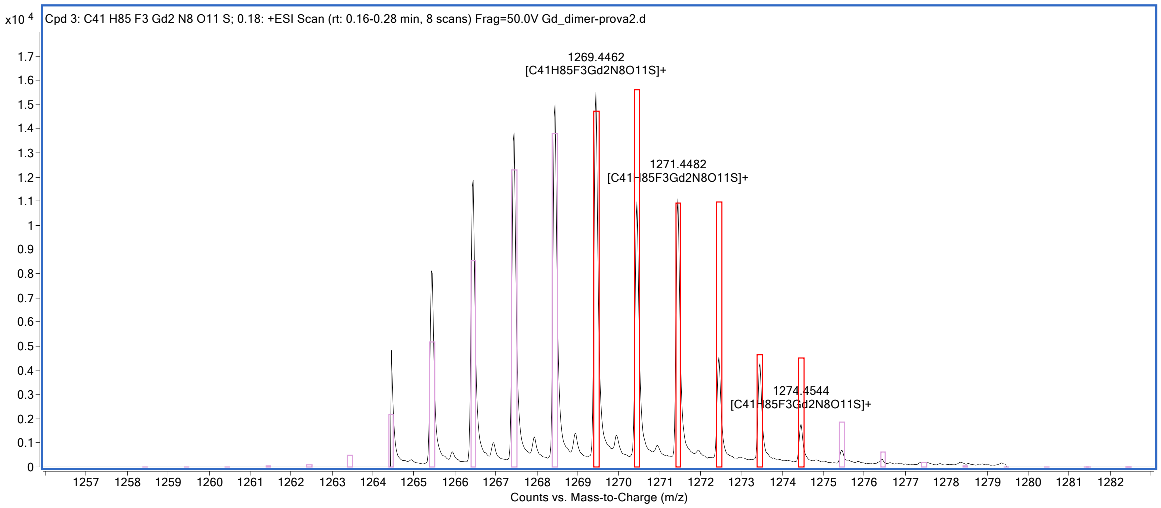

Figure S3. HRMS spectra of the Gd(THP) dimer with the triflate ion. The experimental spectrum is shown in black, while the red bars represent the calculated isotopic distribution for the ion $[C_{41}H_{85}F_3Gd_2N_9O_{11}S]^+$.

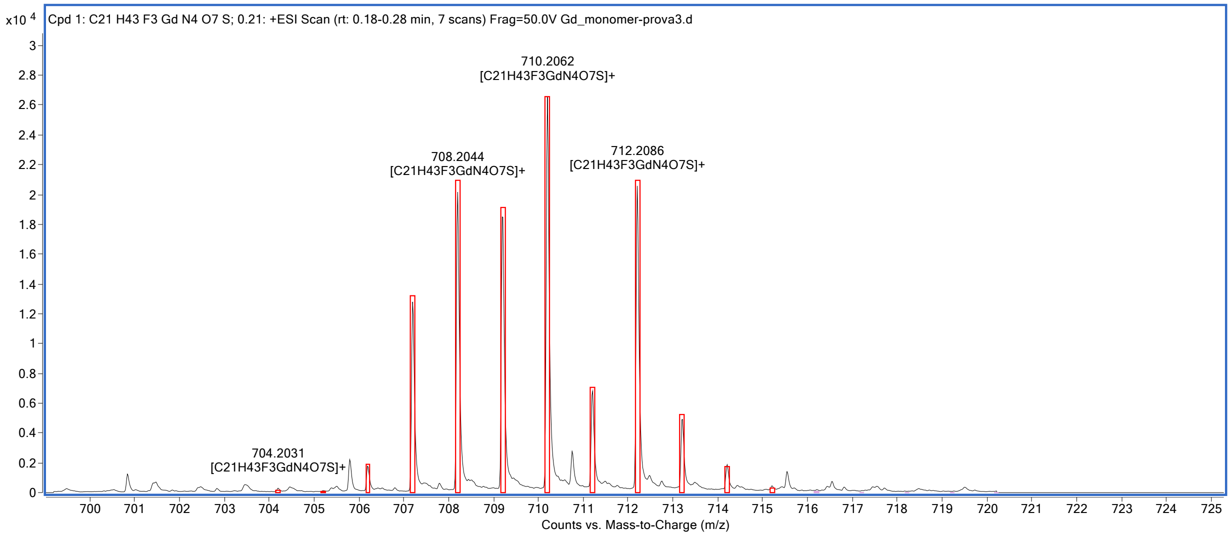

Figure S4. HRMS spectra of the Gd(THP) monomer with the triflate ion. The experimental spectrum is shown in black, while the red bars represent the calculated isotopic distribution for the ion $[C_{21}H_{43}F_3GdN_4O_7S]^+$.

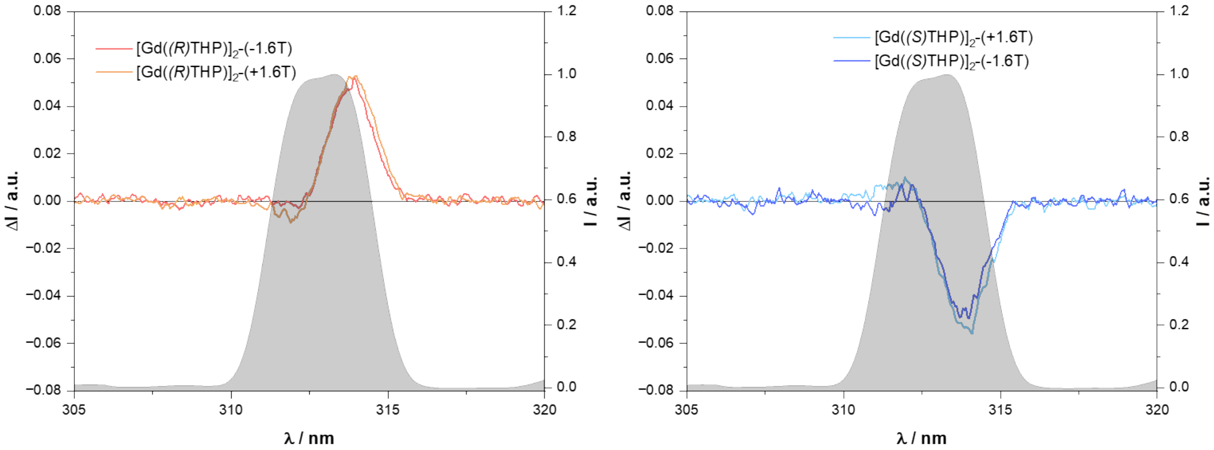

Figure S5. Superposition of the CPL and emission spectra recorded in acetonitrile (C ≈ 11 mM) with both orientations of the magnet for [Gd(*(S)*THP)]$_2$ and [Gd(*(R)*THP)]$_2$.

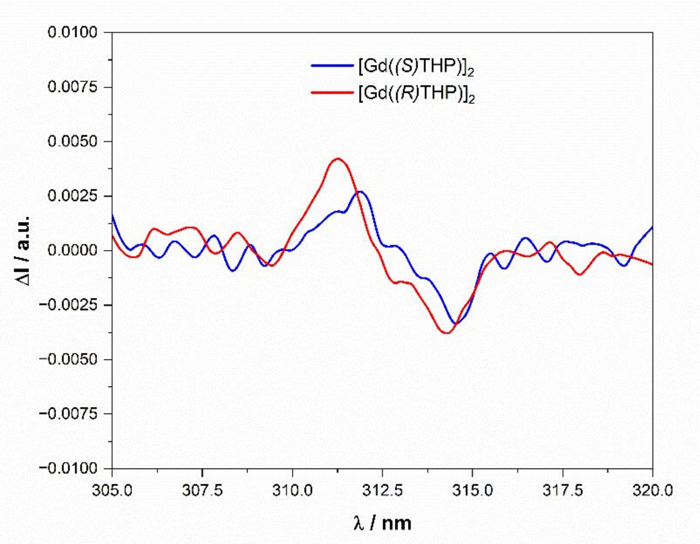

Figure S6. Superposition of the MCPL components for both enantiomers of [Gd(THP)]$_2$ recorded in acetonitrile (C ≈ 11 mM).

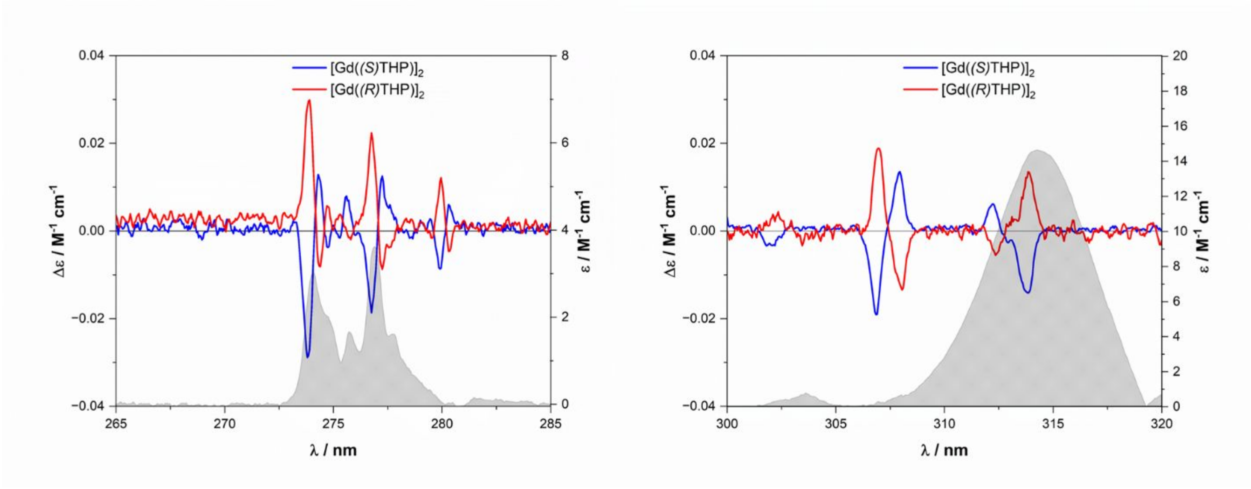

Figure S7. ECD spectra of $Gd(THP)_2$ in ACN (C ≈ 11 mM) The corresponding absorption spectra are traced in background.

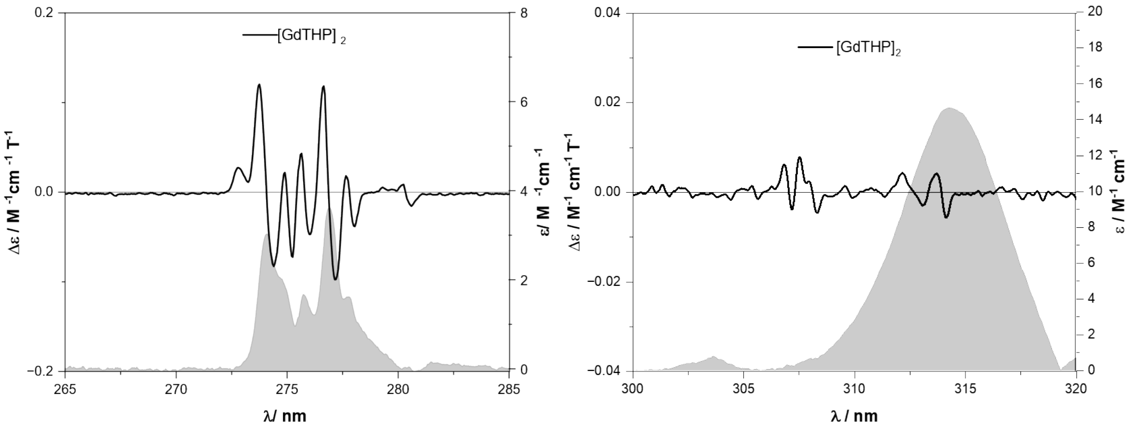

Figure S8. MCD and absorption spectra of $[Gd(THP)]_2$ in ACN (C ≈ 11 mM).

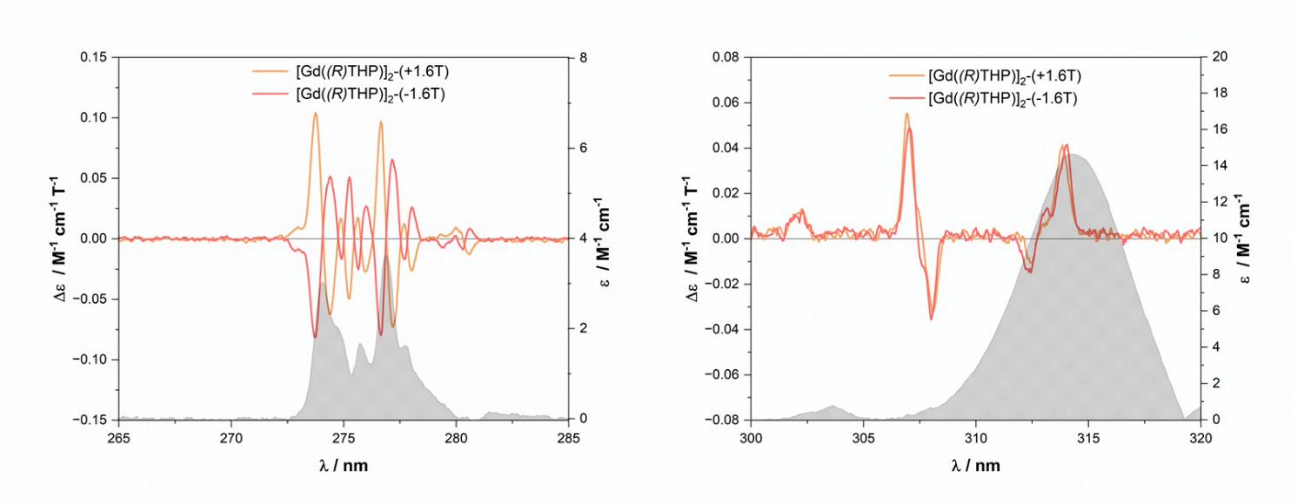

Figure S9. Superposition of the CD and absorption spectra recorded in ACN (C ≈ 11 mM) with both orientations of the magnet for $[Gd((R)THP)]_2$.

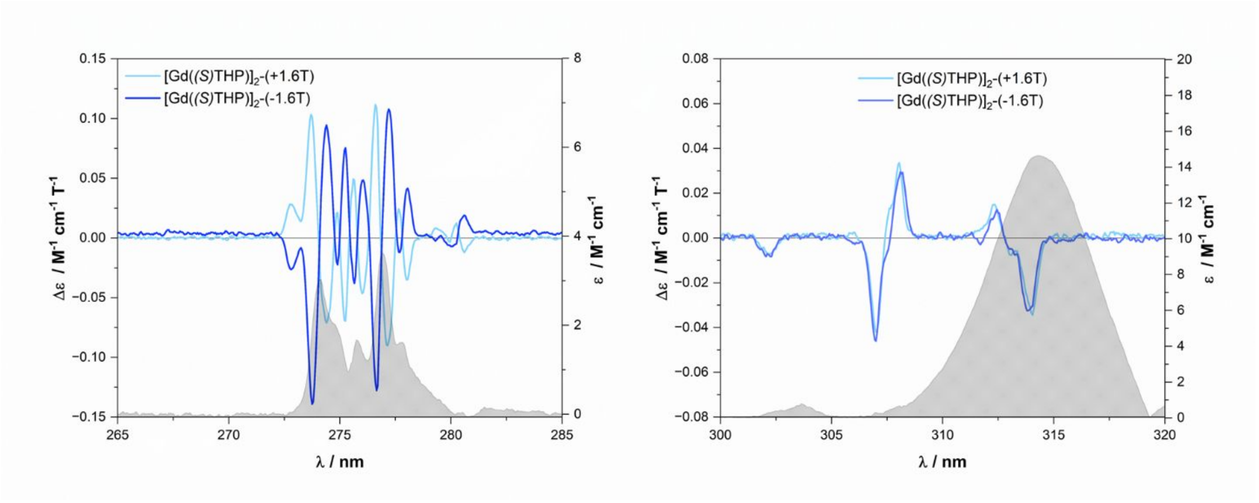

Figure S10. Superposition of the CD and absorption spectra recorded in (C ≈ 11 mM) with both orientations of the magnet for $[Gd((S)THP)]_2$.

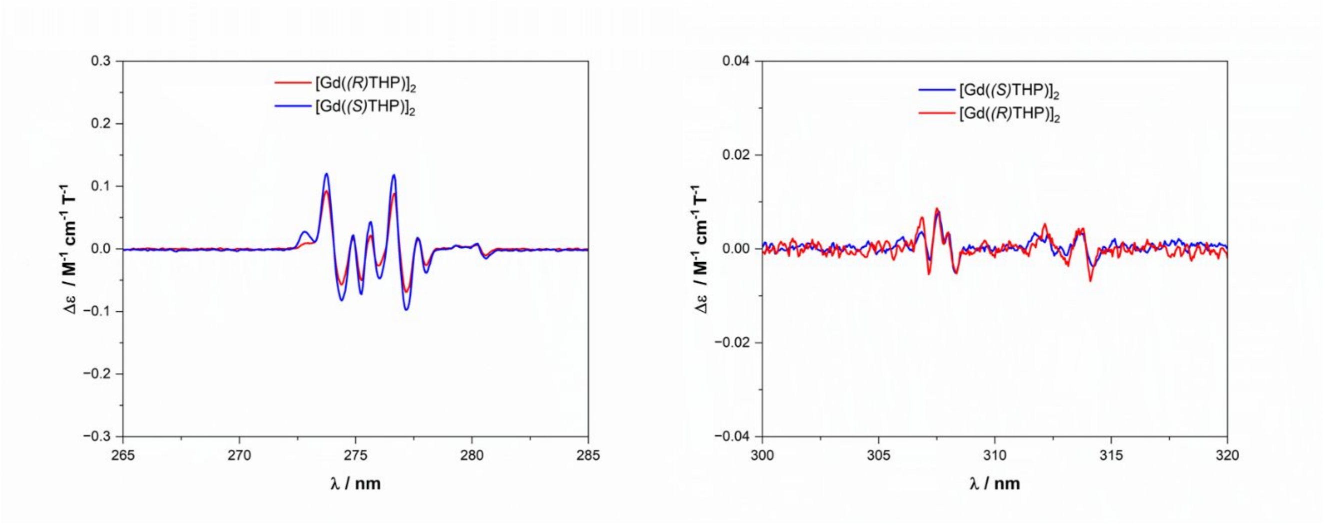

Figure S11. Superposition of the MCD components for both enantiomers of $[Gd(THP)]_{2.}$ recorded in ACN (C ≈ 11 mM).

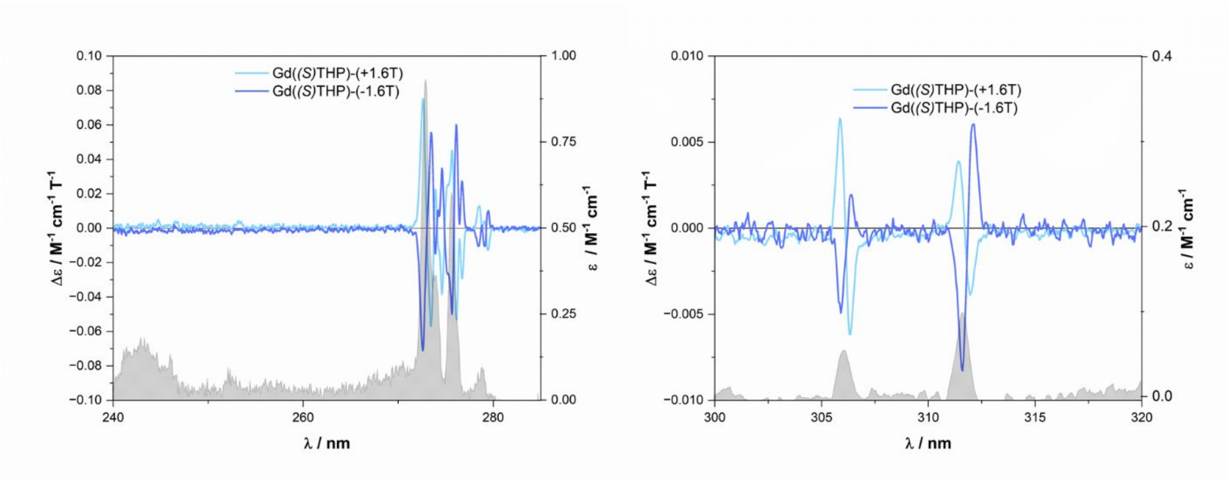

Figure S12. Superposition of the CD and absorption spectra recorded in water (pH<1,C ≈ 27 mM) with both orientations of the magnet for Gd(*(S)*THP) monomer.

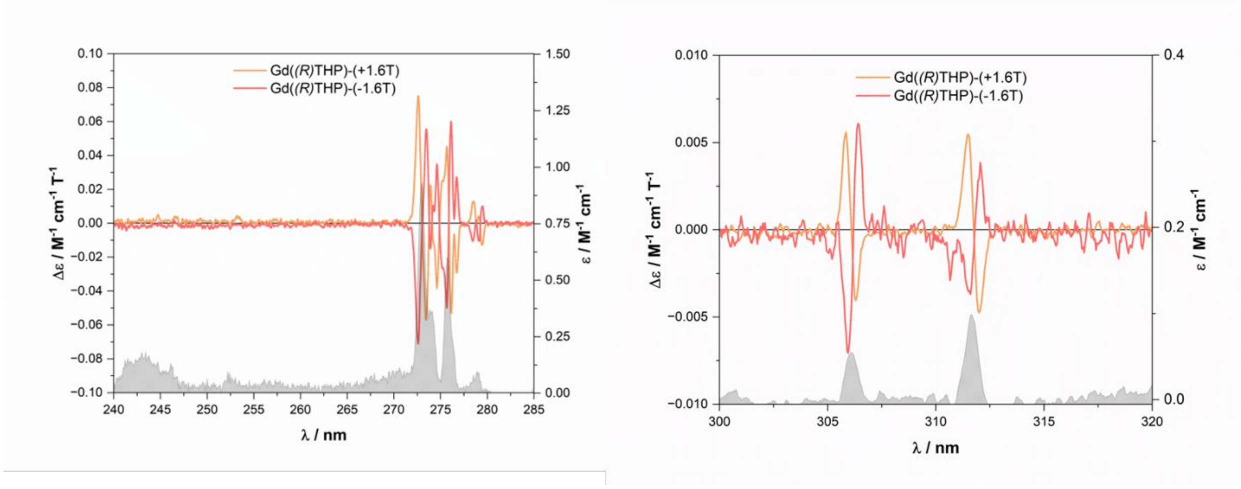

Figure S13. Superposition of the CD and absorption spectra recorded in water (pH<1,C ≈ 27 mM) with both orientations of the magnet for Gd(*(R)*THP) monomer.

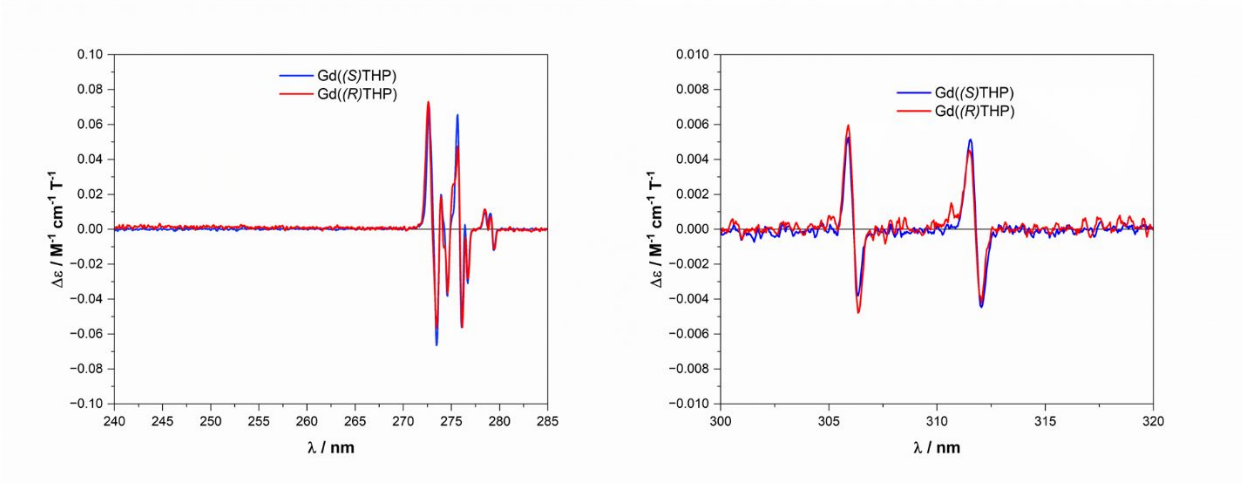

Figure S14. Superposition of the MCD components for both enantiomers of Gd(THP) recorded in water (pH<1,C ≈ 27 mM).

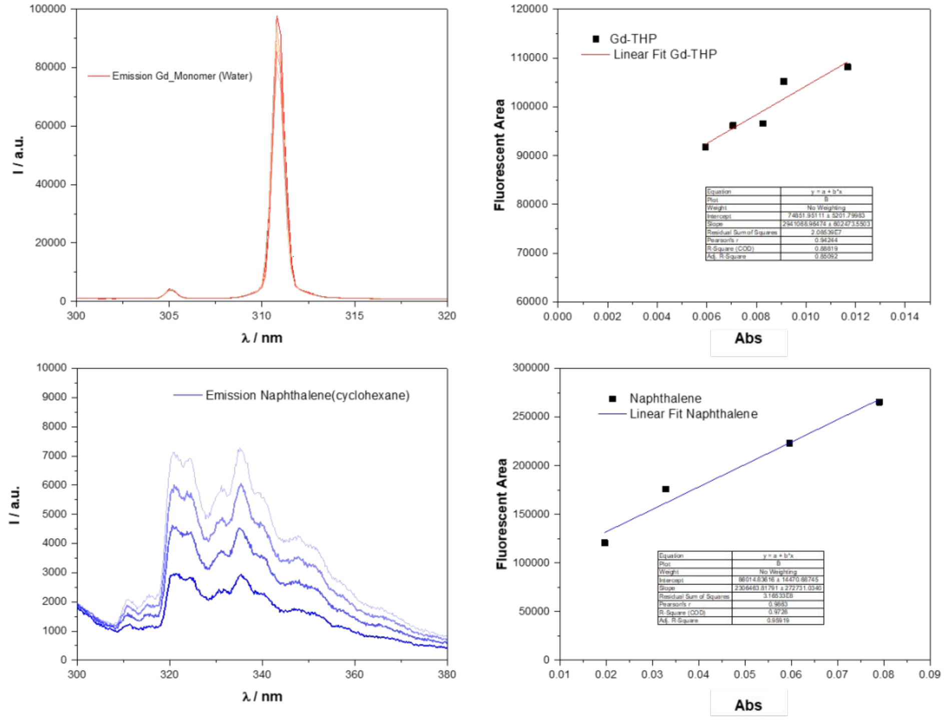

Figure S15. Emission spectra recorded at increasing concentrations for Gd(THP) monomer (red) and naphthalene (blue), with the corresponding linear fits of integrated emission intensity (right panels).

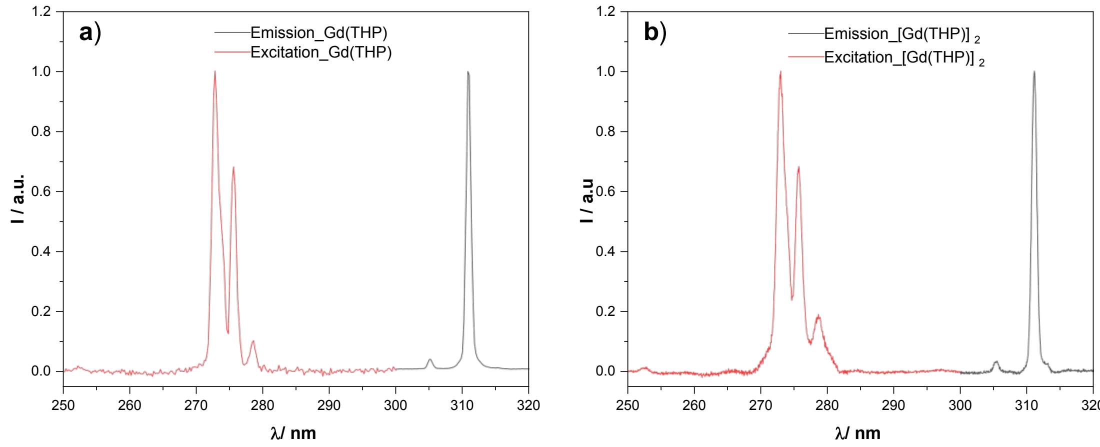

Figure S16. Emission and Excitation spectra of a) Gd(THP) 6 mM in H_2O (pH<1) and b) $[Gd(THP)]_2$ in ACN (8mM)

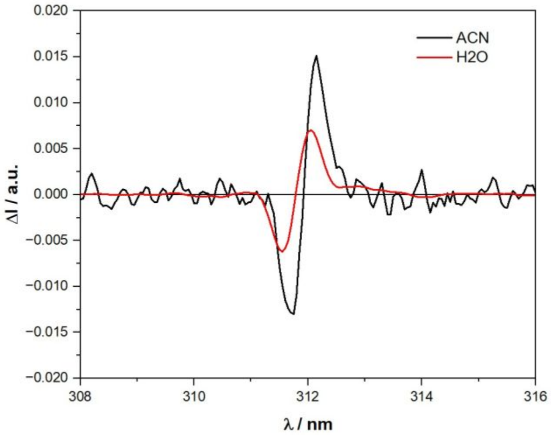

Figure S17. Superposition of the CPL spectra of Gd(*S*)THP in ACN and H_2O (pH<1).

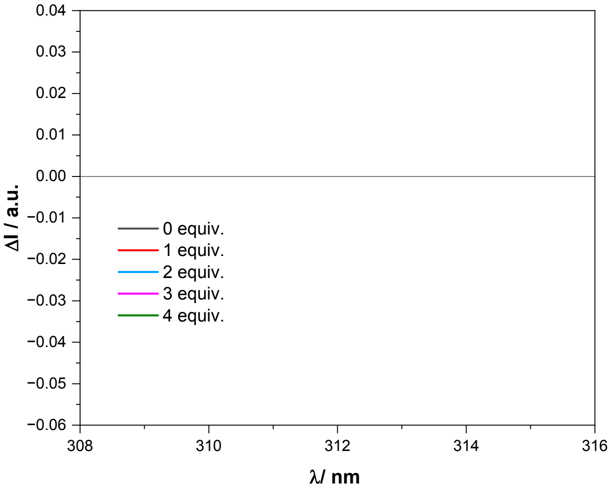

Figure S18. CPL spectra of Gd((S)THP) in ACN (6 mM) upon increasing quantities of Et3N.

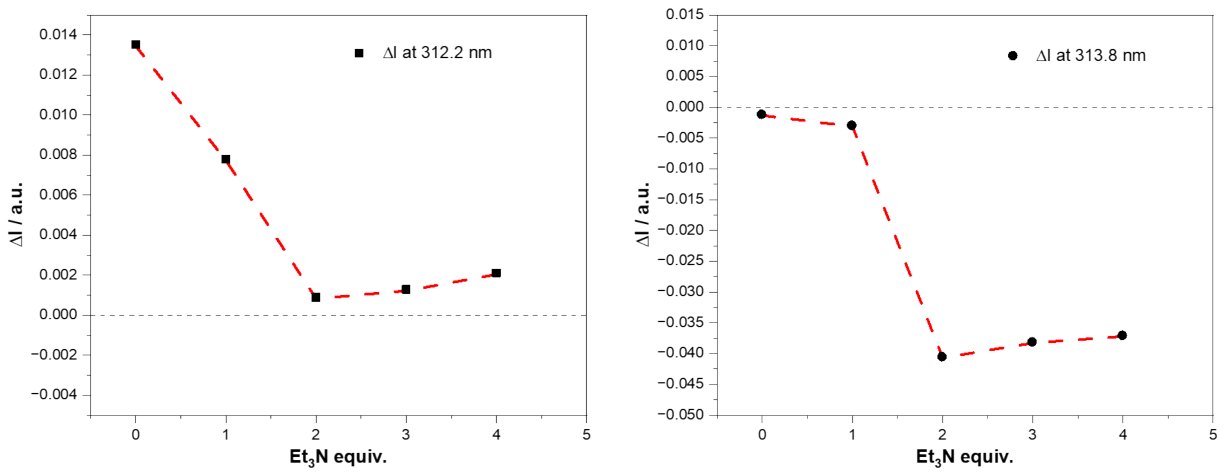

Figure S19. Evolution of the CPL signal upon addition of Et3N. Left: variation of CPL signal at 312.2 nm. Right: variation of CPL signal at 313.8 nm.

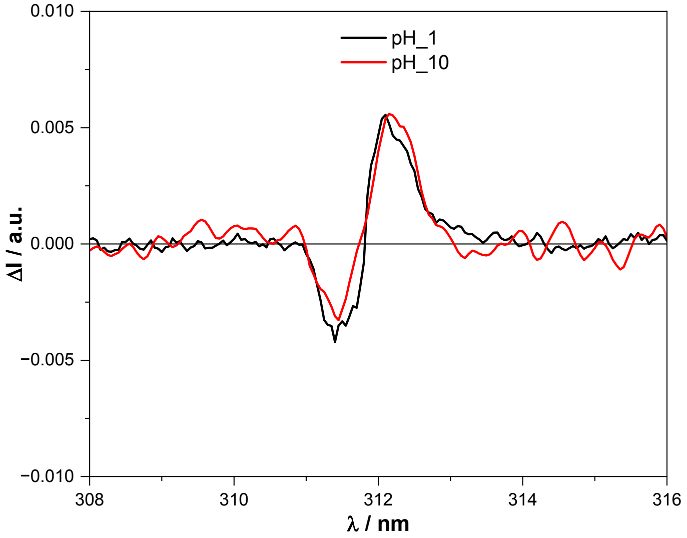

Figure S20. CPL spectra of Gd((*S*)THP) in water (10mM) at different pH values.

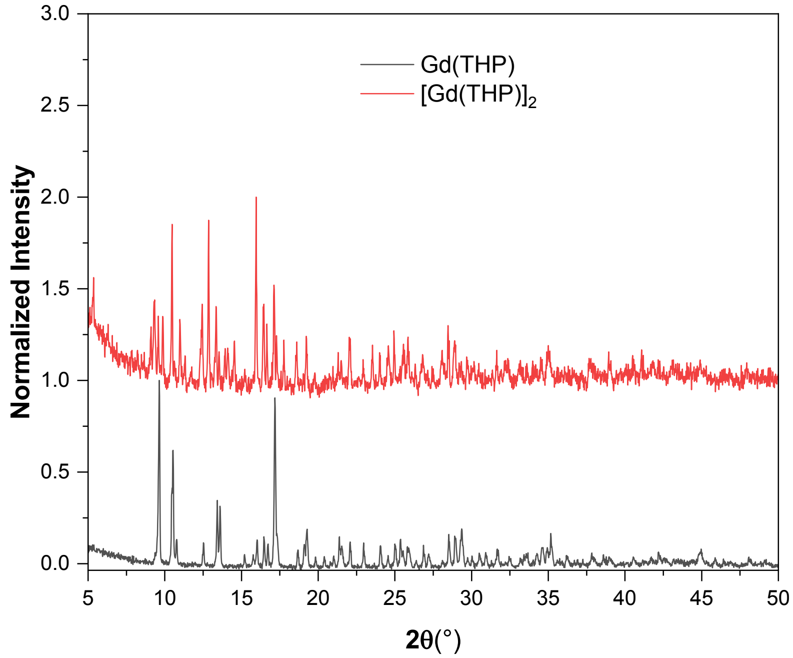

Figure S21. PXRD spectrum of Gd(THP) and $[Gd(THP)]_2$

4 NMR Spectra

^1H NMR spectra of THP ligand

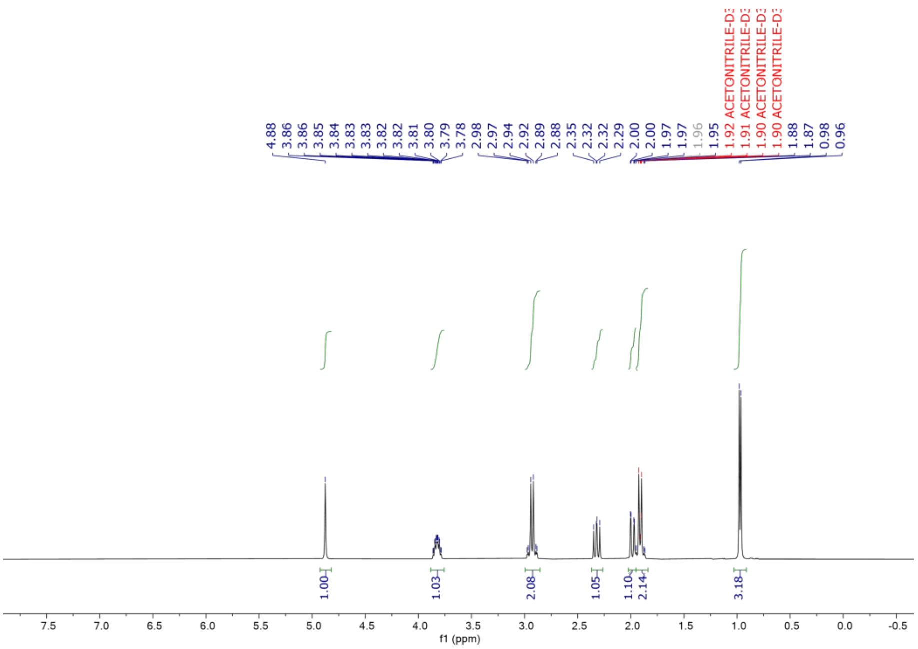

^{13}C NMR spectra of THP ligand

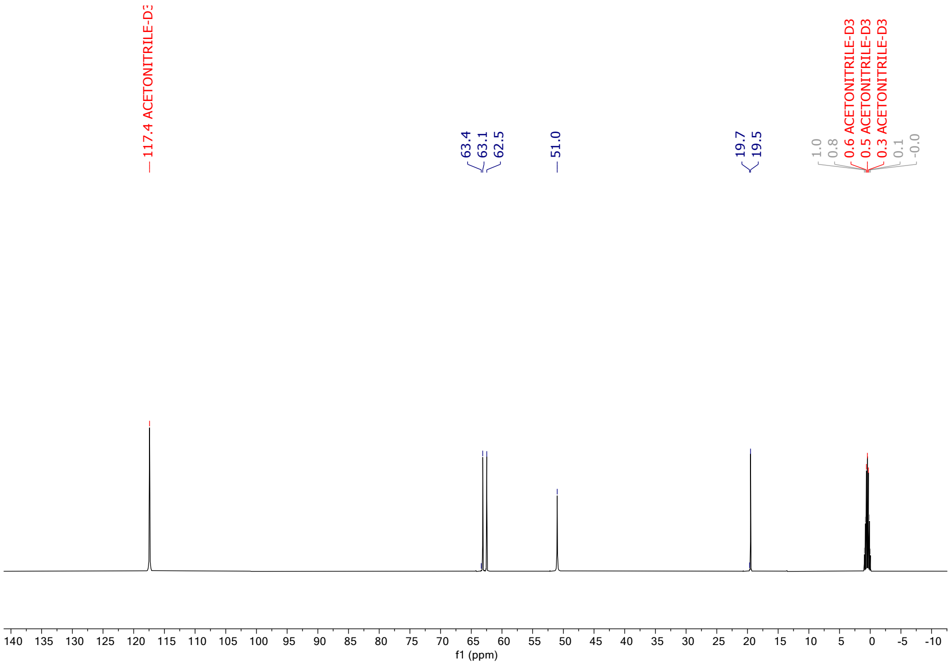

5 References

(1) Aileen Chin, K.O., Morrow, J.R., Lake, C.H., Churchill, M.R. Synthesis and solution properties of lanthanum(III), europium(III), and lutetium(III) THP complexes and an x-ray diffraction study of a crystal containing four stereoisomers of a europium(III) THP complex (THP = 1,4,7,10-tetrakis(2-hydroxypropyl)-1,4,7,10-tetraazacyclododecane). Methyl groups impart rigidity to S,S,S,S-THP macrocyclic complexes. *Inorganic Chemistry* **1994**, *33* (4), 656-664.

(2) Lelli, M.; Di Bari, L. Solution structure and structural rearrangement in chiral dimeric ytterbium(III) complexes determined by paramagnetic NMR and NIR-CD. *Dalton Transactions* **2019**, *48* (3), 882-890, DOI: 10.1039/C8DT03090A.
